# Effectiveness of *ab initio* molecular dynamics in simulating EXAFS spectra from layered systems

**DOI:** 10.1107/S1600577524005484

**Published:** 2024-07-23

**Authors:** F. d’Acapito, M. A. Rehman

**Affiliations:** a Consiglio Nazionale delle Ricerche, Istituto Officina dei Materiali – OGG, c/o ESRF, Grenoble, France; b Department of Chemical and Materials Engineering, New Uzbekistan University, Tashkent, Uzbekistan; ESRF – The European Synchrotron, France

**Keywords:** EXAFS, *ab initio* molecular dynamics, density functional theory, thin films

## Abstract

The simulation of EXAFS spectra of thin films via *ab initio* methods is discussed. The procedure for producing the spectra is presented as well as an application to a two-dimensional material (WSe_2_) where the effectiveness of this method in reproducing the spectrum and the linear dichroic response is shown. A series of further examples in which the method has been employed for the structural determination of materials are given.

## Abstract 2

*Ab initio* molecular dynamics has been used to study the structure of a WSe_2_ multilayer deposited on silicon.

## 1. Introduction

The study of functional materials requires detailed knowledge of the structure of the systems under investigation. X-ray absorption spectroscopy (XAS), and in particular extended X-ray absorption fine structure (EXAFS), are powerful techniques that can provide a clear description of the local surrounding of selected chemical species contained in the material under study. It is not usually sufficient to obtain bond distances and the nature of the first neighbours from EXAFS; rather, a detailed description of the site of the component under study is necessary. EXAFS can provide a detailed picture of the local structure around a chemical species within a distance of a few angstroms. When carrying out data analysis it is necessary to compare the experimental data with theoretical hypotheses about the site and in most cases experimental reference compounds are not available. For this, the use of *ab initio* structural simulations can be helpful, as modern density functional theory (DFT) methods allow structures to be simulated to an accuracy of a few % for cell dimensions (Csonka *et al.*, 2009 ▸) or interatomic distance values that match well with the accuracy of the EXAFS. In several cases, a simple comparison between the numerical results from DFT (namely, shell radii) is not sufficient due to the complexity of the site under study. If we consider a dopant in a matrix there are several possibilities for its incorporation site: substitutional, interstitial, coupled to vacancies, *etc*. These structures can be simulated but, in order to assess the occurrence of one of them in the sample under study, a description of the site with several coordination shells is necessary. This would be complex to carry out using classical methods (too many parameters like *N*, *R* and σ^2^ for the many shells to compare numerically) whereas direct comparison with the related simulated spectra could easily solve the task. Indeed, a site can be identified by the various features that appear in the spectrum and that result from the complex superimposition of all the (most significant) scattering paths of the photoelectron. The problem is that for a reliable calculation the dynamics of the structure under study also need to be considered as the damping of EXAFS single contributions critically depends on atomic motions and their mutual correlations.

Molecular dynamics (MD) has been used for simulating EXAFS data since the pioneering works (D’Angelo *et al.*, 1994 ▸; Palmer *et al.*, 1996 ▸; Kuzmin *et al.*, 1997 ▸) on ions in liquid that were based on classical MD using interatomic pair potentials. This approach is particularly effective in terms of the required computational resources, and permits the use of cells containing hundreds of atoms and long thermalization times (tens of picoseconds). On the other hand, the availability of appropriate pair potentials is needed or even their optimization/refinement on the system under study (Kuzmin & Evarestov, 2009 ▸; Anspoks *et al.*, 2012 ▸), and the level of agreement between theory and experiment is remarkable.

For treating the general case, *ab initio* DFT simulations can be used by various approaches. A method has been proposed (Vila *et al.*, 2007 ▸, 2008 ▸, 2012 ▸, 2018 ▸) that theoretically calculates the Debye–Waller factors of the paths to be used for the simulation of the EXAFS spectrum with a considerable level of success (Veronesi *et al.*, 2010 ▸). Another approach consists of running an *ab initio* molecular dynamics (AIMD) cycle and then calculating the EXAFS spectra from a series of snapshots of the cycle (Bocharov *et al.*, 2016 ▸, 2020 ▸). A comparison of *ab initio* generated EXAFS spectra can also be used to validate *ad hoc* generated potentials as shown by Shapeev *et al.* (2022 ▸). Also, in these cases the quality of reproduction of the experimental data can be impressive even if the computational resources needed are considerable. MD can also be used to simulate theoretical spectra from tentative structures to interpret experimental data. In this case a close agreement is not required, as long as the candidate structures bear clear differences and the requirements on computation resources can be relaxed. An example of a structural choice made by comparing experiment and AIMD has been given by Cartechini *et al.* (2011 ▸) where the focus of the study was the incorporation site of Zn in lead antimonate used for the production of the widespread historical paint pigment *Giallo Napoli*. The EXAFS spectrum at the Zn *K*-edge [see Fig. 6(*a*) of Cartechini *et al.* (2011 ▸)] showed features that are well reproduced by the simulated spectrum of Zn substituting for Sb and were in anti-phase with respect to the simulated spectrum of Zn substituting for Pb. This showed a counter-intuitive result, as it would be expected that the divalent Zn ion would substitute for the divalent Pb rather than Sb. Further examples of structural determination obtained from comparison of experimental EXAFS spectra and AIMD have been given by d’Acapito *et al.* (2016 ▸) regarding the incorporation site of Er^3+^ ions in CaF_2_ crystals and by Kopula Kesavan *et al.* (2020 ▸) regarding the local structure around Ag in Ag_*x*_Bi_1–*x*_S_2_ nanoparticles.

Regarding layered systems, an example of an investigation has been given by d’Acapito *et al.* (2020 ▸), focusing on the structure of [(GeTe)_2_/(Sb_2_Te_3_)_*m*_]_*n*_ superlattices used for an innovative class of materials for non-volatile memories called interface phase change random access memories (IPCRAMs). In particular, the debate concerned the real structure (*i.e.* piling order of Ge, Te and Sb layers) occurring in the system among the different ones proposed in the literature (Tominaga *et al.*, 2014 ▸) and on the possible presence of inter-diffusion in the GeTe layer with Sb/Ge substitutions (Casarin *et al.*, 2016 ▸). The method was first validated by reproducing the EXAFS spectrum of the GeTe bulk structure which revealed a considerably close agreement. Then, the structures proposed were simulated by AIMD, and the related calculated EXAFS spectra were compared with experiment [see supplementary Fig. S9 in d’Acapito *et al.* (2020 ▸)]. A candidate structure could be identified but the agreement was still not satisfactory due to the presence of intermixing. As a second step, an inter-diffused structure (obtained by randomly exchanging Ge with Sb) was created and a second EXAFS spectrum was derived that better matched the experiment with a 30% intermixing. Due to strong intermixing and the loss of good order in the direction perpendicular to the surface, no dichroic analysis was carried out in that case.

In the present contribution, AIMD has been used to interpret data on a well oriented sample of WSe_2_ consisting of a few layers deposited on a silicon substrate. The dichroic signal has been computed and compared with experiment with good agreement, showing that AIMD can be effectively used on surface systems.

## 2. Computation strategy

In order to reproduce the EXAFS spectra the associated structural simulation needs to be based on *ab initio* methods, both for the static and for the dynamic part. This could be done in principle with appropriate pair potentials for selected cases but these are not widely available, and for a general approach it is more advisable to adopt the *ab initio* method. In the examples that follow, the simulations were carried out at the ESRF user’s computing cluster (https://www.esrf.fr/Infrastructure/Computing/NICE/Implementation) using the *VASP* code (Kresse & Furthmüller, 1996 ▸) with the PBE-sol functional (Csonka *et al.*, 2009 ▸), non-relativistic calculations and unpolarized spin densities. A model of the structure under investigation is created first and a static DFT relaxation is carried out in order to deal with a cell that already possesses a minimized energy. This step can be carried out with a small cell, and a 10 × 10 × 10 *k* mesh in the Brillouin zone is used. Once relaxed, a supercell is created in order to start the AIMD cycle. The size of the supercell is critical, as on one hand it would be desirable for it to be as large as possible to simulate the EXAFS spectrum with the longest paths possible, but on the other the use of *ab initio* methods requires a considerable computational effort and, apart from using dedicated machine-time on supercomputing centres, cells beyond 150 atoms can barely be treatable on smaller machines. It is worth pointing out that the maximum distance that can be reproduced in the EXAFS spectrum is half the size of the supercell, as beyond that value replicas of the same atoms in the structure will create unphysically intense signals. The supercells of the examples presented here have a size of 6–10 Å and 80–120 atoms. The AIMD cycle is carried out in DFT mode at the Γ point using the same functional as for the static relaxation and a Nosé–Hoover thermostat (Nosé, 1984 ▸; Hoover, 1985 ▸) for controlling the temperature. This parameter requires some attention as AIMD is intrinsically semi-classic and does not take into account zero-point vibrations. To treat this aspect, a useful rule-of-thumb can be derived from the discussion presented by Kuzmin (2017 ▸) and based on the result of Yang & Kawazoe (2012 ▸) on monoatomic lattices where it was found that the amplitude of the zero-point vibrations equals that of the excited vibrations at a temperature *T*_0_ corresponding to about one-third of the Debye temperature *T*_D_. For reproducing spectra of common crystals at room temperature (RT) or above with *T*_D_ of the order of 300–500 K (namely, Cu, Ge, Zn) the actual temperature can be used. For the same systems when reproducing spectra collected at liquid-nitrogen temperature (LNT) it is more appropriate to use a somewhat higher value in the calculation, ideally 1/3*T*_D_. However, when a close agreement between simulation and experiment is needed at low temperature the path-integral Monte-Carlo/MD approach is recommended (Kuzmin *et al.*, 2016 ▸; Beccara & Fornasini, 2008 ▸).

In this contribution theoretical spectra were generated using the *Feff8* code (Ankudinov & Rehr, 2000 ▸) with self-consistent calculation of the electron density (self-consistent field cluster of about 40 atoms corresponding to a radius of 4–5 Å depending on the cases) and using the Hedin–Lunqvist exchange correlation potential for the inelastic effects. The full calculation of the electron density is carried out on the initial structure, stored and used for all the other frames. Considering that the atomic oscillations around the equilibrium positions are small with respect to the interatomic distances at the temperature value considered, this density function can be safely transferred to all the structures in the sequence. For building up the EXAFS signal, multiple-scattering events of up to four legs for the photoelectron were considered and the maximum path length was set at half of the supercell in order to avoid strong signals generated by atomic replicas in the periodic cell. Global amplitude 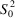 and edge energy position *E*_0_ were left at the values determined by the calculation.

A short description of the codes developed for extracting the instantaneous structures from the MD cycles and calculating the EXAFS averages has been given by Cresi (2015 ▸).

## 3. Examples

### 3.1. A benchmark system: metallic Cu

**Figure 1:**
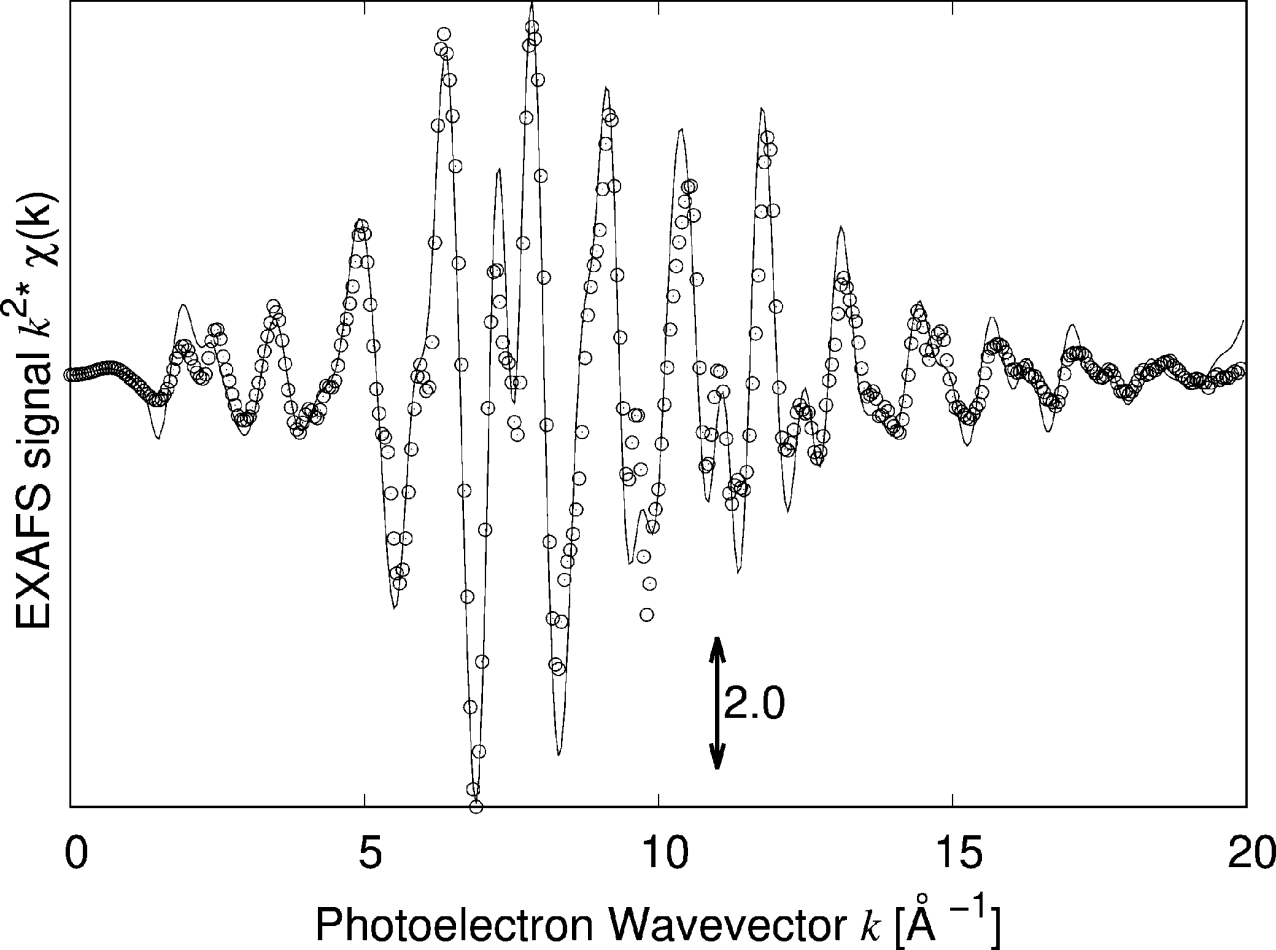
Comparison of the experimental (circles) and simulated (line) EXAFS spectra of metallic Cu at LNT. The lattice parameter of the theoretical cell was expanded by 1% before calculating the EXAFS spectra in order to better show the agreement with the experimental data. It is worth noting that 1% is a typical absolute inaccuracy of DFT calculated structures.

In this section an example of EXAFS spectra calculated *ab initio* on a well known system will be given, in order to assess the reliability of the method. The aim of this step is to verify that the overall shape and features of the spectrum can be reproduced with the tentative structure (face-centred cubic Cu in this case) and with the methods chosen (the PBE-sol functional). The scope here is to have available a method that permits the EXAFS data to be related to one structure among several, as shown by d’Acapito *et al.* (2020 ▸). Obtaining a close agreement between experiment and theory making use of advanced and resource-consuming methods is a successive step in this analysis that is not considered at this stage. All experimental EXAFS spectra presented hereafter have been collected at the LISA beamline at the European Synchrotron Radiation Facility (d’Acapito *et al.*, 2019 ▸). For the simulation a cubic supercell of 108 atoms (10.7 Å in size) derived from a static relaxed cell was used for the AIMD cycle at 150 K. Time-steps of 3 fs were used and frames were saved every three time-steps. A first cycle of 1.8 ps was used for thermalization and a successive cycle of 1.8 ps was used for the EXAFS simulation obtained with the last 0.9 ps (100 spectra). This time duration is limited by the computation resources available. However, it corresponds to several typical atomic oscillation periods, whose value is around 10^−13^ s, so provides a fair estimation of the signal damping, although remaining a limiting factor of the correct reproduction of the details of the signal amplitude. The convergence of the simulation was checked by looking at the residual ξ, defined as the sum of the difference of the squared spectra Θ_*N*_(*k*) obtained for *N* and*N* − 1 frames,
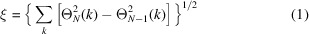
(d’Acapito *et al.*, 2016 ▸) and was found to be a few 10^−3^ units (see supporting information). Figs. 1 ▸ and 2 ▸ show the results.

The features of the spectrum in *k* space, namely the amplitude and position of the crests and valleys of the oscillations (bearing information about amplitude and phase of the signals) as well as the peaks in the Fourier Transform are correctly reproduced, meaning that the method can simulate both the structure and the associated dynamics fairly well though with a less accurate agreement on the signal damping at high *k* values that nonetheless does not prevent the recognition of the phase. A more accurate modelling of the dynamics would lead to a better agreement (see Shapeev *et al.*, 2022 ▸) but would require larger cells and a more sophisticated description of the interatomic potentials that is beyond the scope of this contribution.

### 3.2. A real-life system: two-dimensional WSe_2_

The case shown above deals with a polycrystal, thus providing a spherically averaged spectrum. However, XAS is a polarization-dependent technique and useful information can be derived by exploiting the linear dichroism of XAS when applied to non-cubic systems. Here, the case of multilayers (about three or four) of WSe_2_ deposited on Si for opto-electronic applications is presented. WSe_2_ represents an interesting family of two-dimensional materials and has been proposed as a component of photosensitive hetero-junctions (Choi *et al.*, 2020 ▸) stacked with MoSe_2_ or Mo_*x*_W_1–*x*_Se_2_ layers. The samples were grown by using a chemical vapour deposition system. The Si substrates were first cleaned by the RCA cleaning standard method and then placed in a two-inch vertical cold-wall chamber, where WSe_2_ growth was conducted. The W and Se precursors, in the gaseous phase, were fed into the chamber as tungsten hexacarbonyl [THC; W(CO)_6_] and diethyl sulfide [DES; (C_2_H_5_)_2_Se], respectively. The parameters were set for THC at 0°C and for DES at −15°C, for the vapour phase growth of WSe_2_. Additionally, Ar and H_2_ were pumped into the chamber to supply the precursors for Se and W, respectively, and their reaction. A total pressure of 50 Torr, a growth temperature of 600°C and a growth time of 140 min were found to be the ideal experimental parameters. In the case of THC, only 3 s.c.c.m. [where s.c.c.m. is standard cubic centimetres per minute] gas flow was used; however, for the Ar gas it was 10 s.c.c.m. (Rehman *et al.*, 2023 ▸). The layers have been characterized by electron microscopy as shown in the supporting information.

Layered systems of the class (transition-metal)–(chalcogen)_2_ can be effectively studied by comparison of the MD-simulated spectra and EXAFS. In a recent paper, Pudza *et al.* (2023 ▸) studied the MoS_2_ system in powdered samples. The comparison between experimental and theoretical EXAFS spectra obtained by MD and reverse Monte Carlo methods permitted deep details on the intra- and inter-layer structures to be retrieved as well as the mean-square displacements Mo–O and Mo–S as a function of temperature.

This topic can be extended by noting that the hexagonal structure (typical of these systems) leads to a strongly (linear) dichroic XAS signal that can be exploited to better reveal possible interactions with nearby layers or with the substrate. This method of data collection can be realized only on samples in thin-film monocrystalline form and addresses aspects that play a crucial role in improving the performance of opto­electronic devices. In the present study we have investigated whether dichroic signals can be effectively simulated by *ab initio* methods. Measurements were carried out at RT at the Se *K*-edge with the beam polarization vector parallel or perpendicular to the sample surface, *i.e.* with respect to the hexagonal plane of the sample. EXAFS data were collected using an Si(111) monochromator and Si-coated mirrors for harmonic rejection. The sample was placed at grazing incidence with respect to the incoming beam at an angle of ∼0.1° in both cases and data were collected in fluorescence mode using a four-element silicon drift detector (Hafizh *et al.*, 2019 ▸).

**Figure 3:**
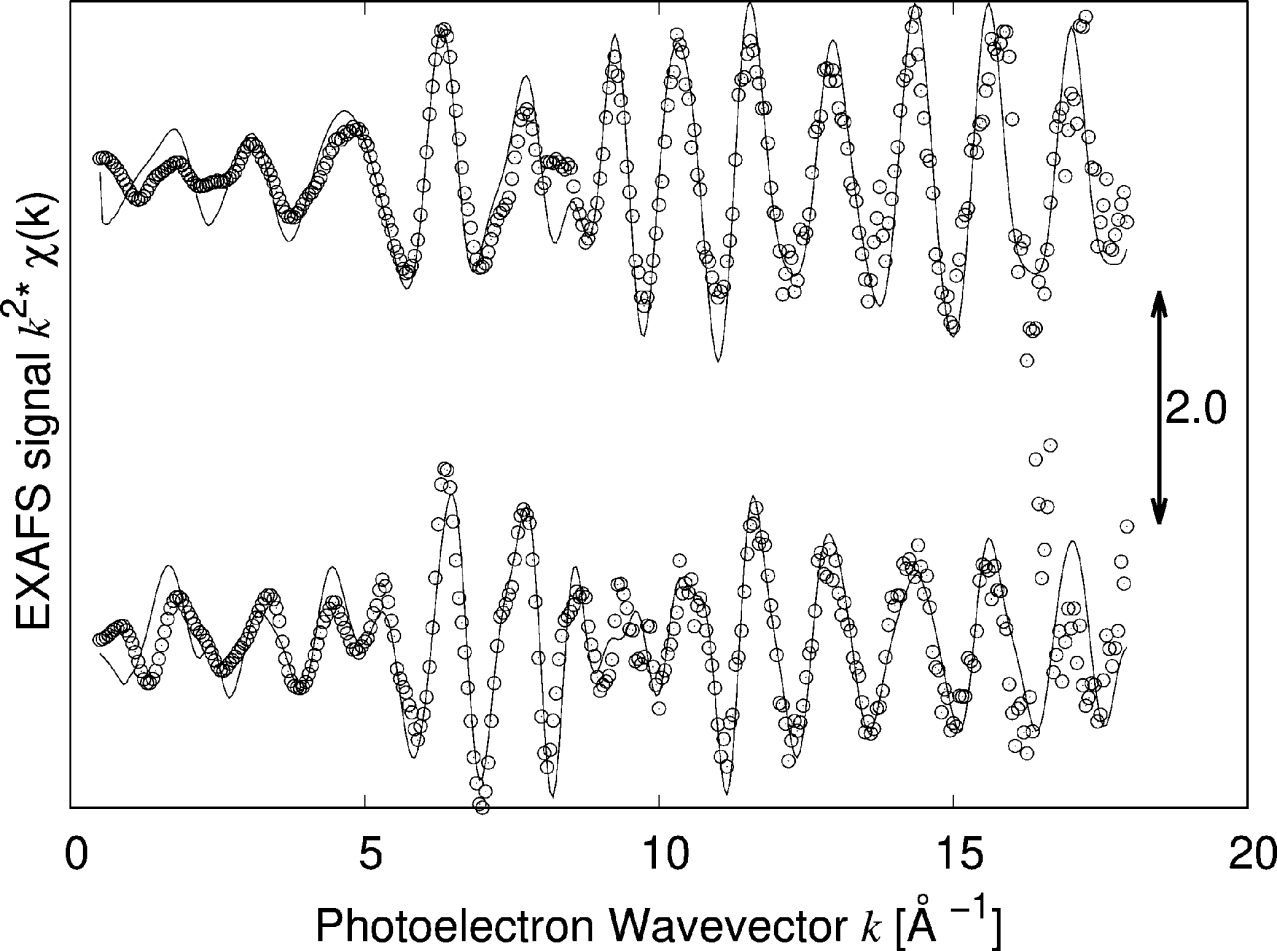
Comparison of the experimental (circles) and simulated (line) EXAFS spectra at the Se *K*-edge of WSe_2_ at RT with the polarization parallel (bottom) and perpendicular (top) to the surface.

The EXAFS data were analysed with the support of AIMD simulations at RT carried out on a structure consisting of a single 2D layer Se–W–Se in a lattice of 13 Å × 13 Å in the plane and a vacuum separation of 25 Å (see supporting information) derived from the structure presented by Schutte *et al.* (1987 ▸). This model mimics a free-standing layer, *i.e.* a layer with a negligible interaction with the substrate. A first cycle for thermalization (6 ps) was followed by a successive 3 ps cycle for calculating the EXAFS. 300 spectra were averaged in this case for a total time of 900 fs and they were calculated for a linear polarization either parallel or perpendicular to the surface. In the first case XAS spectra were calculated with the polarization vector parallel to the *c*-axis; for the latter case a circular average on the *a*–*b* plane was carried out using the related tools available in the *Feff8* code. The results are shown in Figs. 3 ▸ and 4 ▸.

**Figure 5:**
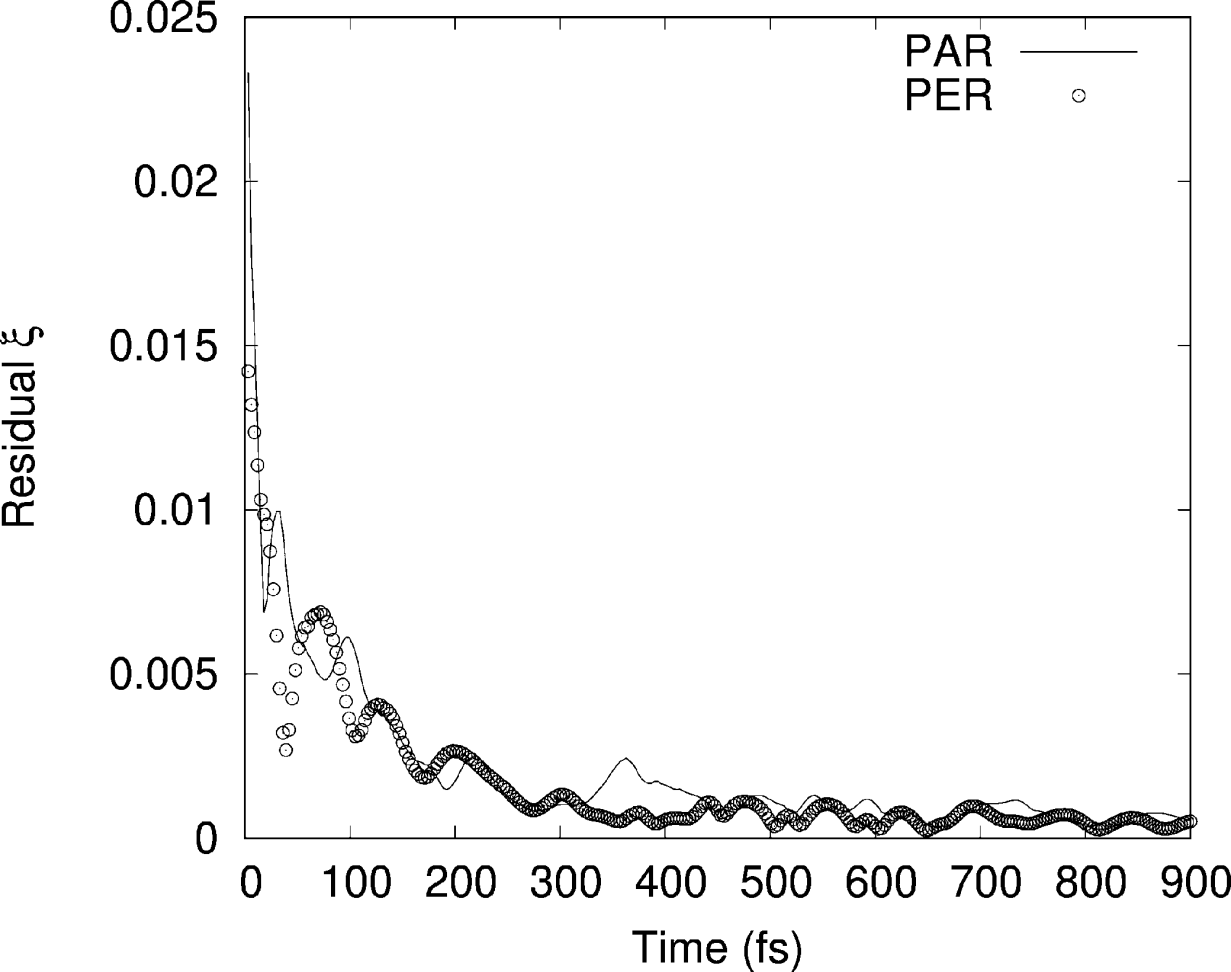
Residual ξ as defined in equation (1) for the PAR and PER simulated XAS spectra of WSe_2_.

The residuals have been calculated using equation (1) and are shown in Fig. 5 ▸. The value of the residuals saturates at an oscillating behaviour at about 300 spectra before the maximum number of elements on average demonstrating the conversion of the procedure. A good agreement between theory and experiment is visible in Fig. 3 ▸, showing that by using AIMD it is possible to reproduce not only the spectral features but also the differences in the parallel (PAR) and perpendicular (PER) polarizations. It is worth noting as an example the difference in the PAR and PER spectra in *k* space at around 5 Å^−1^ where a single peak present in the PER spectrum that splits in two in the PAR spectrum is correctly reproduced by the simulation. The same can be stated for the series of oscillations at around 9 Å^−1^ which exhibit a different amplitude in the PAR and PER spectra that is again well reproduced by the simulation. The convergence of the procedure can be verified by considering the evolution of the residual ξ as a function of the evolution time that reached a minimum in the interval considered here (Fig. 5 ▸). This demonstrates that AIMD can be used not only for reproducing spherically averaged spectra but also for analysing the features derived from the linear dichroic response of the sample. In order to further test the reliability of the simulations a quantitative analysis of the experimental and AIMD-derived spectra in both PAR and PER geometries has also been carried out using theoretical signals from the WSe_2_ structure generated using the *Feff84* code as described previously. Data were reproduced for the first two intra-layer coordination shells, Se–W [*R*_cryst_ = 2.526 Å (Schutte *et al.*, 1987 ▸)] and Se–Se. In the PAR and PER cases the latter corresponded to the Se–Se in-plane coordination (*R*_cryst_ = 3.282 Å) or to the Se–Se out-of-plane coordination (*R*_cryst_ = 3.346 Å). The results are shown in Table 1 ▸. The bond distances in the two datasets compare well and the crystallographic data (Schutte *et al.*, 1987 ▸) and the Debye–Waller factors of the Se—W bond are in good agreement with the determination of Saisopa *et al.* (2023 ▸) (∼0.0030 Å^−2^) carried out at the W *L*_3_-edge.

## 4. Discussion

The present study on layered WSe_2_ shows that AIMD permits the experimental data (containing both the structure and the dynamics at the atomic scale) of the system to be reproduced correctly. The aim here was to correlate a structural model of the experiment and in this case it was possible to confirm the presence of a 2D hexagonal layer. The correct reproduction of the dichroic signal demonstrated that the layer was well oriented on the surface and that there were no detectable traces of inter-diffusion with the substrate or additional phases. A less accurate agreement experiment/simulation is visible in the PER spectrum which can be tentatively explained by the fact that the simulation was a single layer whereas the sample actually consisted of a multilayer that could affect the dynamics of the structure and/or add signals from inter-layer correlations.

This study demonstrates that, by using simple and well established models (in this case the MD–DFT method with the PBE-sol functional), it is possible to simulate with fair accuracy the EXAFS spectrum of a complex structure within some angstroms from the absorber. This is shown to be an effective tool in cases when several candidate structures are proposed for a system [see, namely, Tominaga *et al.* (2014 ▸) and d’Acapito *et al.* (2020 ▸)] and a choice (and possible modifications) is to be made on the bases of the experimental data. This procedure would be extremely complex in a conventional data analysis as several fitting parameters would enter into play making their interpretation difficult. In this formulation the method is oriented to a structural recognition task: once a structure is identified, obtaining its fine details will require more refined simulation methods [see, namely, Pudza *et al.* (2023 ▸)].

This method could be further applied in materials for optoelectronics in studies of stacked layers proposed for the realization of devices (Choi *et al.*, 2020 ▸) in order to reveal proper stacking or possible growth defects. Neat interfaces and obtaining the correct structure are aspects of paramount importance for acquiring the desired electro-optic properties.

## 5. Conclusion

In this study, AIMD has been used to study the structure of a WSe_2_ multilayer deposited on silicon. The Se *K*-edge EXAFS spectra corresponding to two different orientations of the X-ray beam polarization vector (parallel and perpendicular to the sample surface) relating to a free-standing monolayer have been simulated. The simulated spectra show good agreement with the experimental ones, even in the correct reproduction of the signal linear dichroism demonstrating that the layer is well oriented with the *c*-axis perpendicular to the surface and that no interdiffusion occurs with the substrate. This shows how AIMD can be used for validating a structural hypothesis on a material and that, in the case of non-cubic surface systems, the linear dichroism can be effectively exploited.

## Supplementary Material

Supplementary info. DOI: 10.1107/S1600577524005484/up5001sup1.pdf

## Figures and Tables

**Figure 2:**
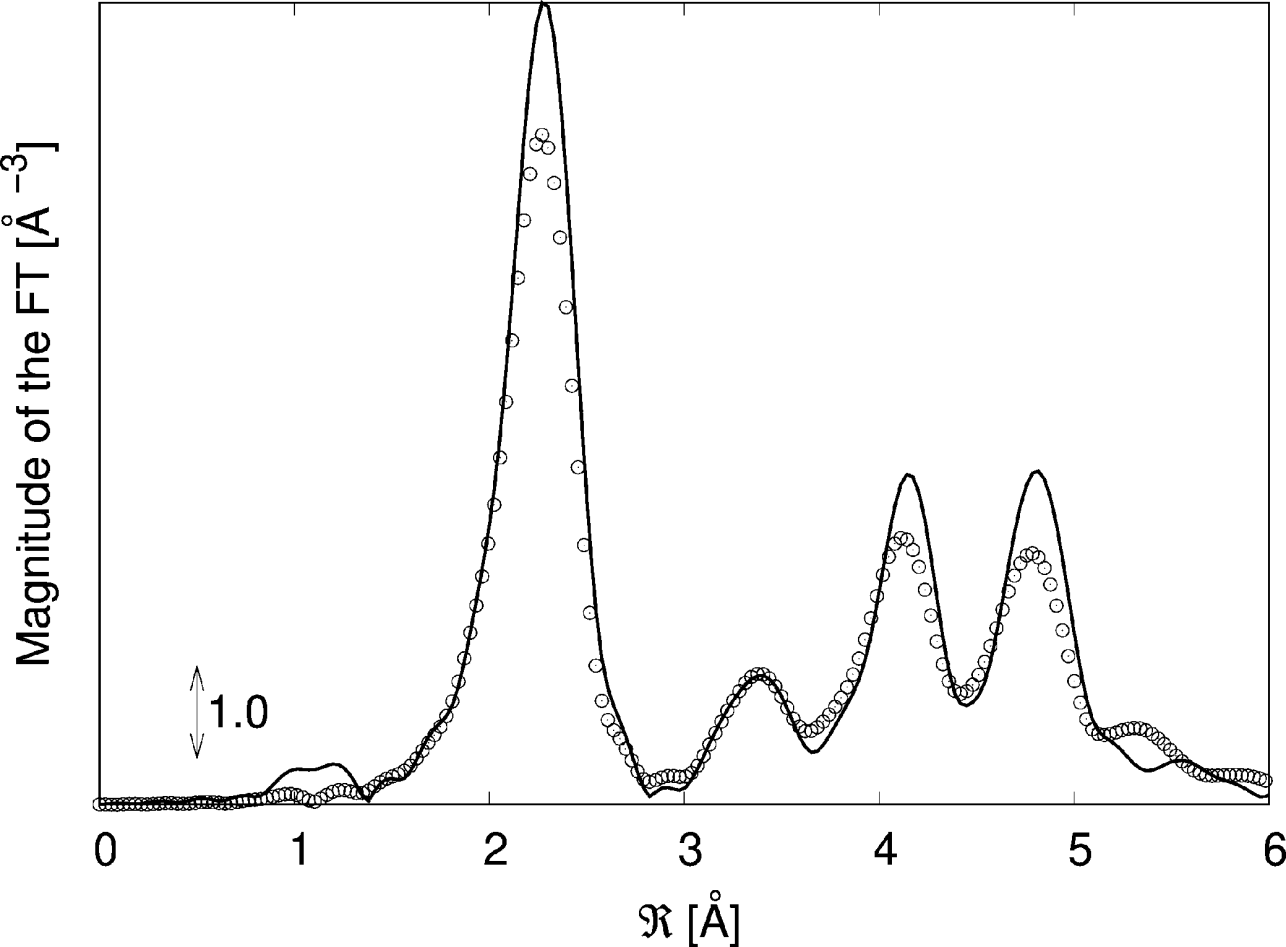
Comparison of the experimental (circles) and simulated (line) Fourier transforms (FTs) of the EXAFS spectra of metallic Cu at LNT.

**Figure 4:**
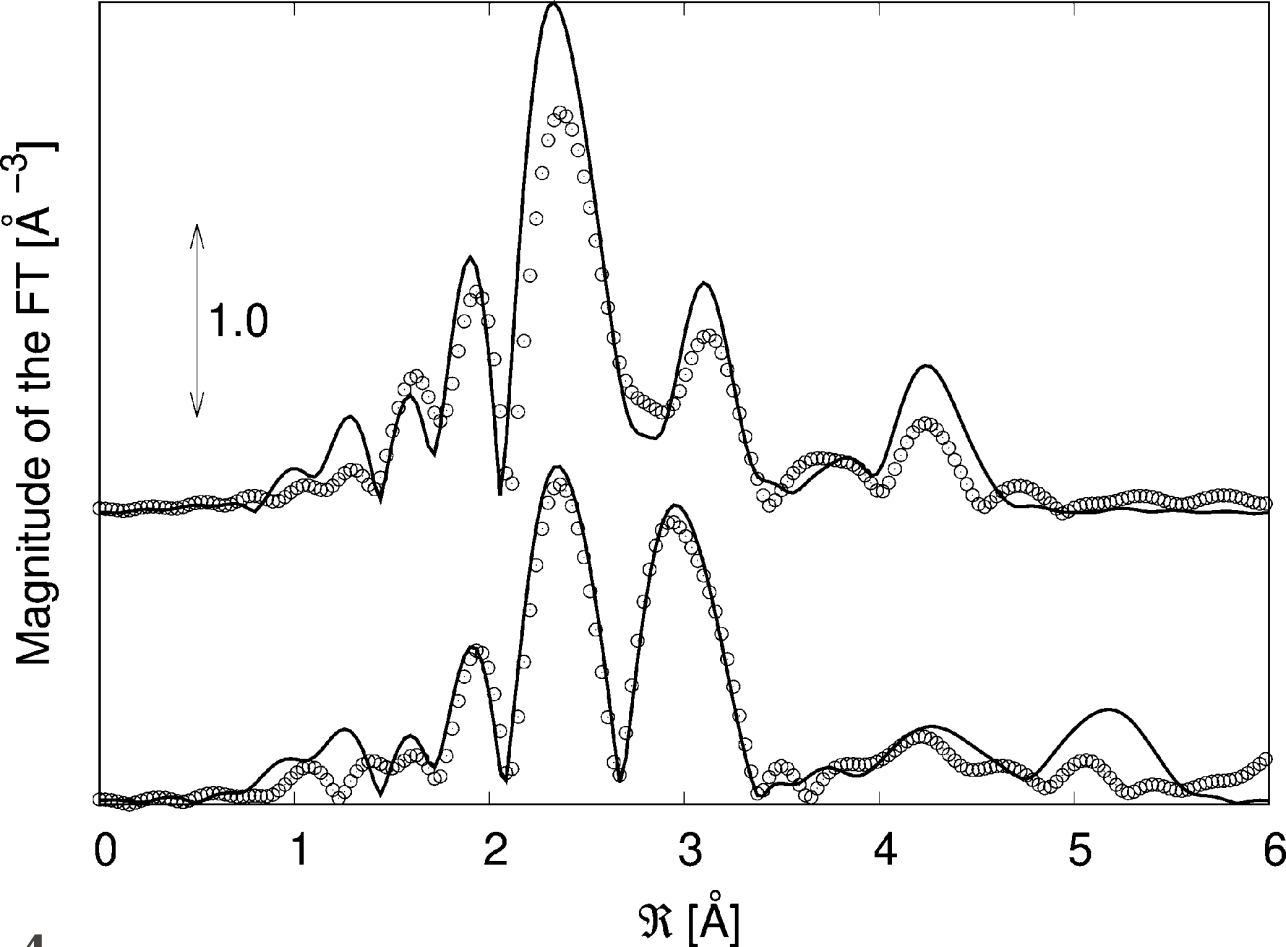
Comparison of the experimental (circles) and simulated (line) FTs of the EXAFS spectra at the Se *K*-edge of WSe_2_ at RT with the polarization parallel (bottom) and perpendicular (top) to the surface.

**Table 1:** Results of the quantitative EXAFS analysis at the Se *K*-edge of the experimental (Exp) and theoretical (Theo) WSe_2_ spectra In all cases the global amplitude reduction factor was found to be 0.92 (5) with the exception of the Exp-PER case where it was 0.7 (1). This could be due to possible bonding with oxygen that was not considered in the fitting model.

Spectrum	*R*_SeW_ (Å)	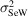 (Å^2^)	*R*_SeSe_ (Å)	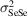 (Å^2^)
Exp-PAR	2.53 (2)	0.0028 (2)	3.28 (2)	0.0085 (4)
Theo-PAR	2.52 (2)	0.0026 (3)	3.27 (3)	0.0082 (7)
Exp-PER	2.52 (2)	0.0026 (3)	3.35 (3)	0.004 (1)
Theo-PER	2.52 (2)	0.0025 (2)	3.34 (3)	0.0032 (3)
