# Supplementary material for: Effectiveness of ab initio molecular dynamics in simulating EXAFS spectra from layered systems
Source: J Synchrotron Radiat. 2024 Jul 23;31(Pt 5):1078–83. doi: 10.1107/S1600577524005484 (PMC11371032; doi:10.1107/S1600577524005484)
Supplement: Supplementary file 1 [file s-31-01078-sup1.pdf]

JOURNAL OF SYNCHROTRON RADIATION

Volume 31 (2024)

Supporting information for article:

Effectiveness of *ab initio* molecular dynamics in simulating EXAFS spectra from layered systems

F. d'Acapito and M. A. Rehman

SUPPLEMENTARY INFO
Effectiveness of *ab initio* Molecular Dynamics in simulating EXAFS spectra from layered systems.

F. d'Acapito

Consiglio Nazionale delle Ricerche, Istituto Officina dei Materiali - OGG, c/o ESRF, Grenoble (France).

E-mail: `francesco.dacapito@cnr.it`

M. A. Rehman

Department of Chemical & Materials Engineering, New Uzbekistan University, Tashkent (Uzbekistan).

1. Introduction

In this document some additional data are presented as a completion of the information presented in the main manuscript.

2. Sample preparation

A Transmission Electron Microscopy image of the sample is shown in Fig. 1: A film consisting in 4 layers of WSe_2 has been created on the surface of the substrate .

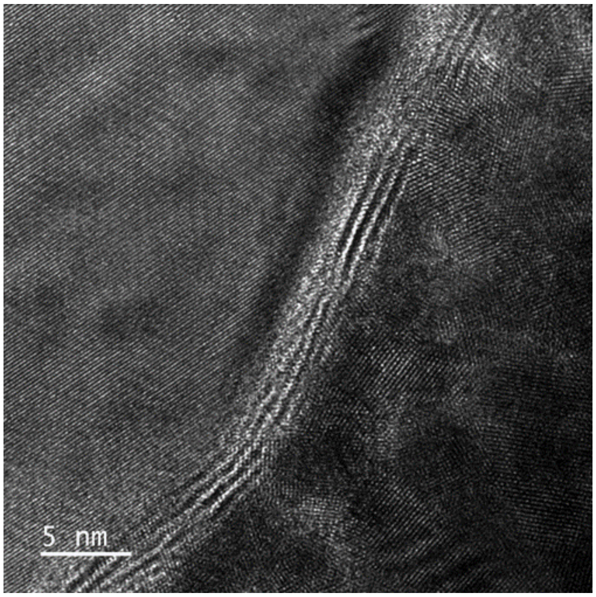

Figure 1. TEM of one specimen of the same preparation batch as the samples studied by XAS.

3. Simulation of the WSe_2 XAFS spectra.

The structure of the WSe_2 layer used in the simulation is shown in Fig. 2: The XAS

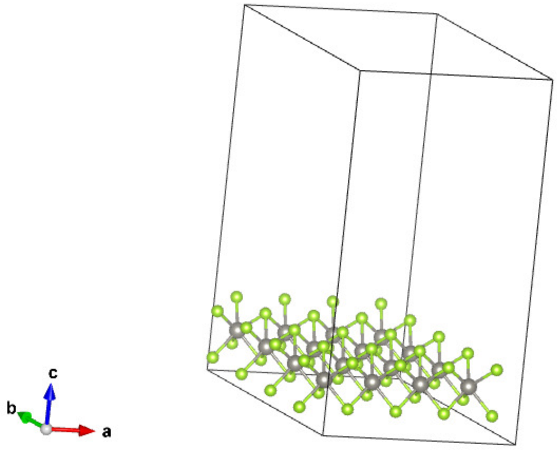

Figure 2. Ball and stick model of the theoretical structure used for the AIMD simulations inserted in the global cell. Green=Se, Gray = W.

data were collected either with the polarization vector parallel to the $a-b$ plane (PAR) or perpendicular to it, along to the c axis (PER).

4. Cu EXAFS spectra simulation

In Fig. 3 presents the evolution of the residual ξ as defined in Eq.1 of the manuscript in the case of Cu

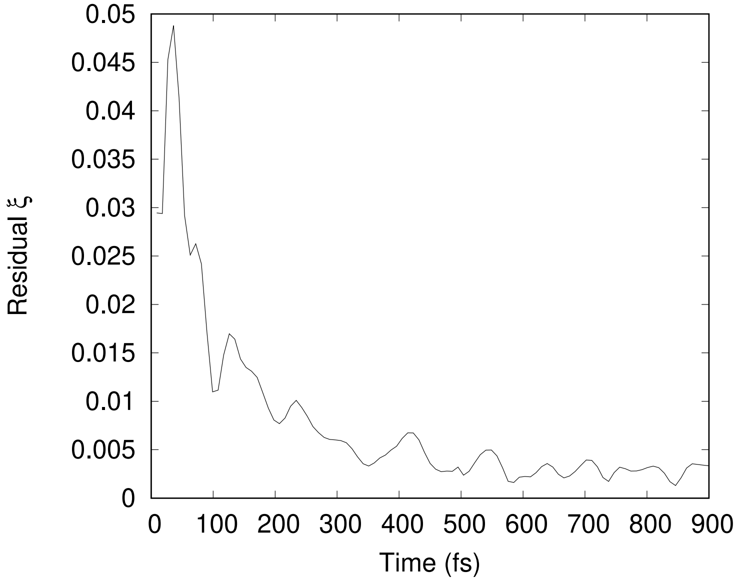

Figure 3.
Residuals of the calculation of the Cu theoretical XAS spectrum. .
